# Supplementary material for: mmsig: a fitting approach to accurately identify somatic mutational signatures in hematological malignancies
Source: Commun Biol. 2021 Mar 29;4:424. doi: 10.1038/s42003-021-01938-0 (PMC8007623; doi:10.1038/s42003-021-01938-0)

Supplementary materials

mmsig: a fitting approach to accurately identify somatic mutational signatures in hematological malignancies

Rustad et al.

Supplementary Figures

Figure S1: Review of mutational profiles reveals clear APOBEC contribution. Three examples where mutational signature profiles can be used to verify the results of mutational signature fitting. **A)** SBS2 (5.7%) and SBS13 (4.9%) were uniquely called by *mmsig* (negative by *deconstructSigs* and *MutationalPatterns*). Both signatures were clearly seen in the mutational profile, with the defining mutational classes identified by arrows. **B)** SBS2 (3.6%) uniquely called by *mmsig* and distinguishable in the mutational profile. **C)** No APOBEC called by either mutational signature fitting algorithm, consistent with absence of distinctive peaks in C>G or C>T.

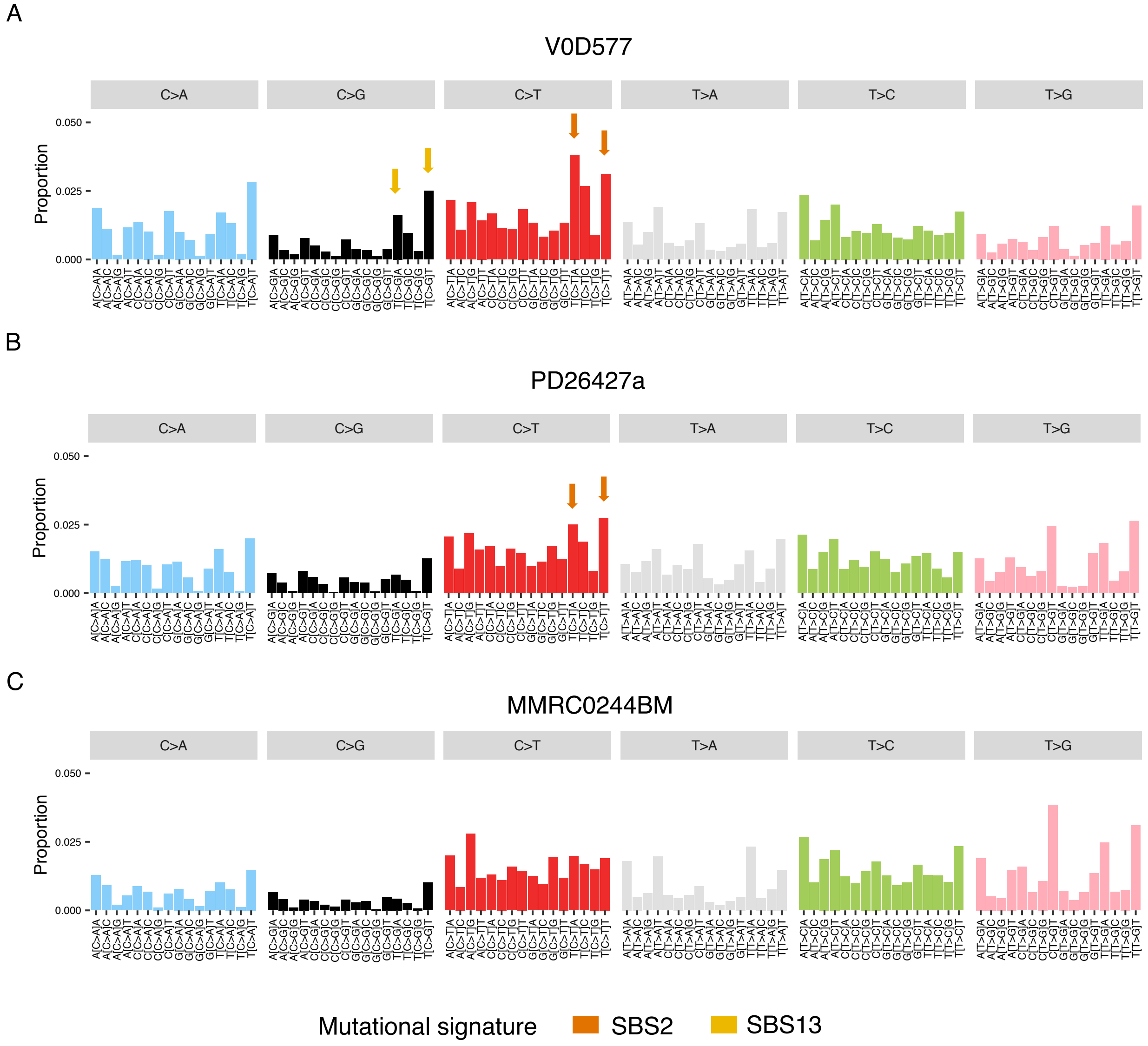

Figure S2: Comparison of new and old COSMIC mutational signature reference. In each panel, the new (i.e. COSMIC v3.1) reference is plotted on the top, the old (i.e. COSMIC v2) reference in the middle, and the difference between the profiles on the bottom. **A)** SBS1. **B)** SBS2

A

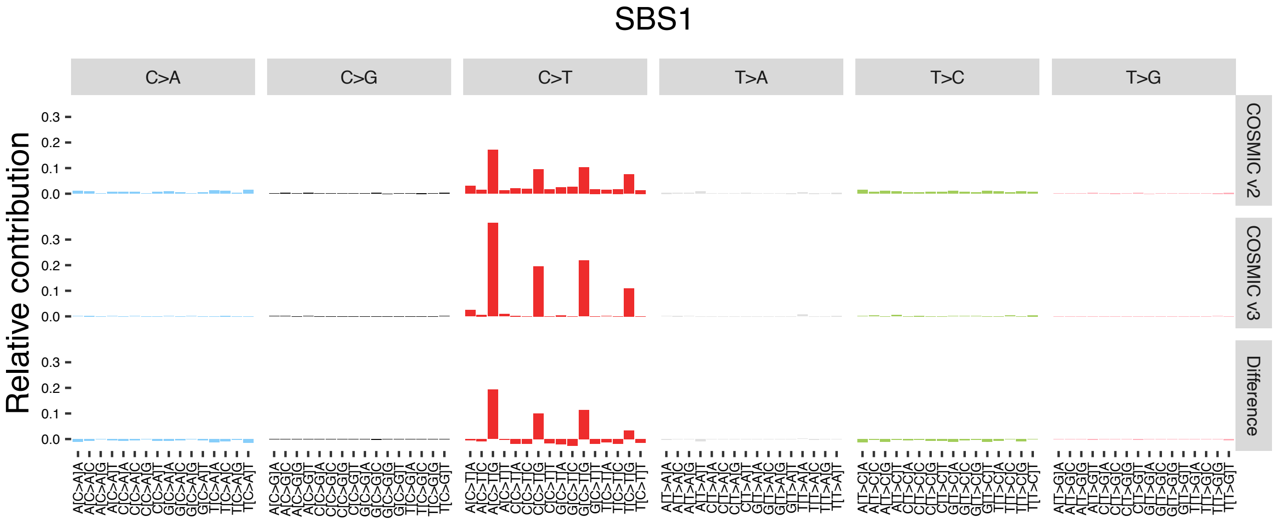

B

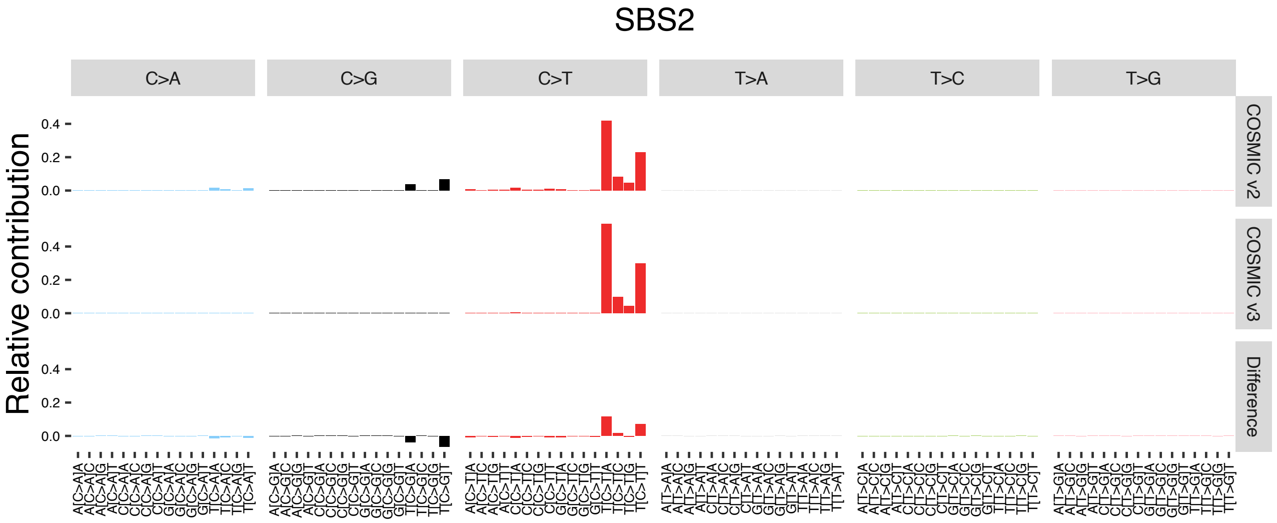

Figure S3: Fitting the COSMIC v2 reference signatures in multiple myeloma. Here, we show the same analysis as in **Figure 2A-B** in the main manuscript but using the COSMIC v2 reference signatures. **A)** Raw algorithm output without any filtering. **B)** Results after applying standard filtering.

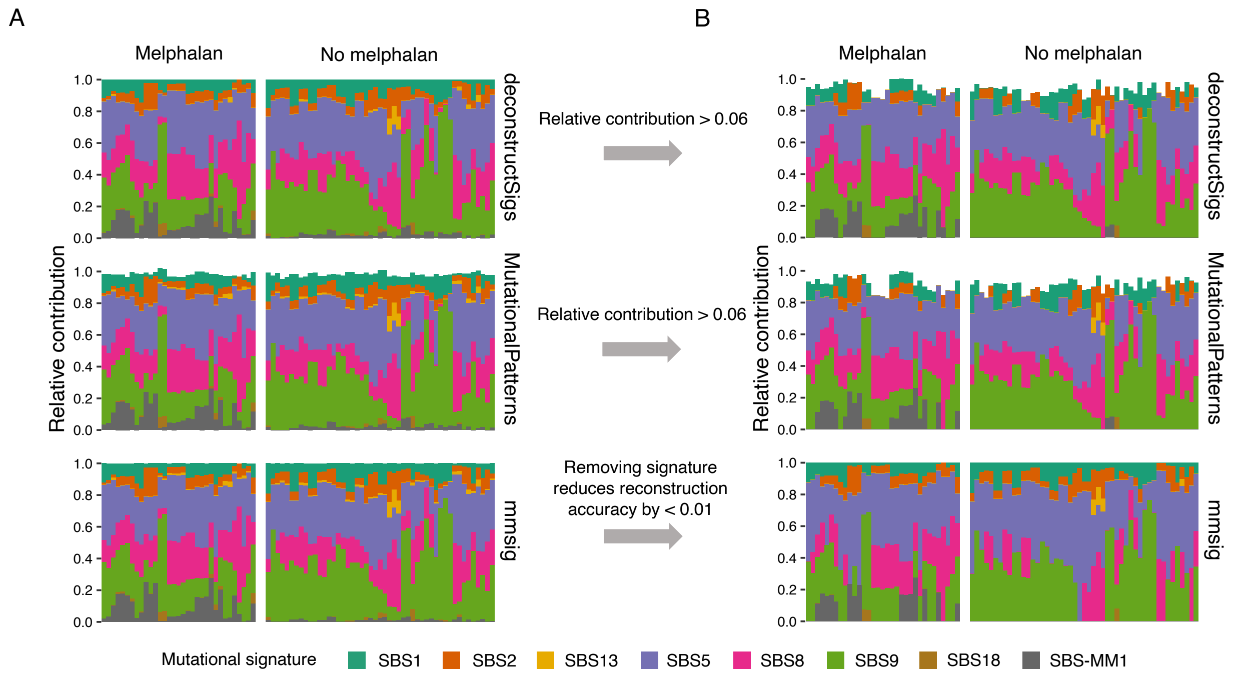

Figure S4: Transcriptional strand bias in patients with and without melphalan exposure. All four samples shown below had statistically significant transcriptional strand bias pooled across the typical mutation types associated with SBS-MM1: C[C>T]A, G[C>T]A, G[C>T]C, G[C>T]G and G[C>T]T. Samples shown in the two upper panels (PD26414g and PD26423h) were exposed to melphalan and had a clear contribution of the SBS-MM1 mutational signature (non-zero 95 % CI). Samples shown in the two lower panels (PD26424a and PD26428c) (two lower panels) were not exposed to melphalan and there was no evidence of the SBS-MM1 mutational signature (the entire 95 % CI was below zero). Taken together, the mutational profiles support the presence of a melphalan footprint in the two upper panels, but not in the lower. Visual inspection also shows a transcriptional strand bias profile more consistent with SBS-MM1 in the upper two panels. A statistical test for transcriptional strand bias provides valuable information, but should be interpreted in light of other data.

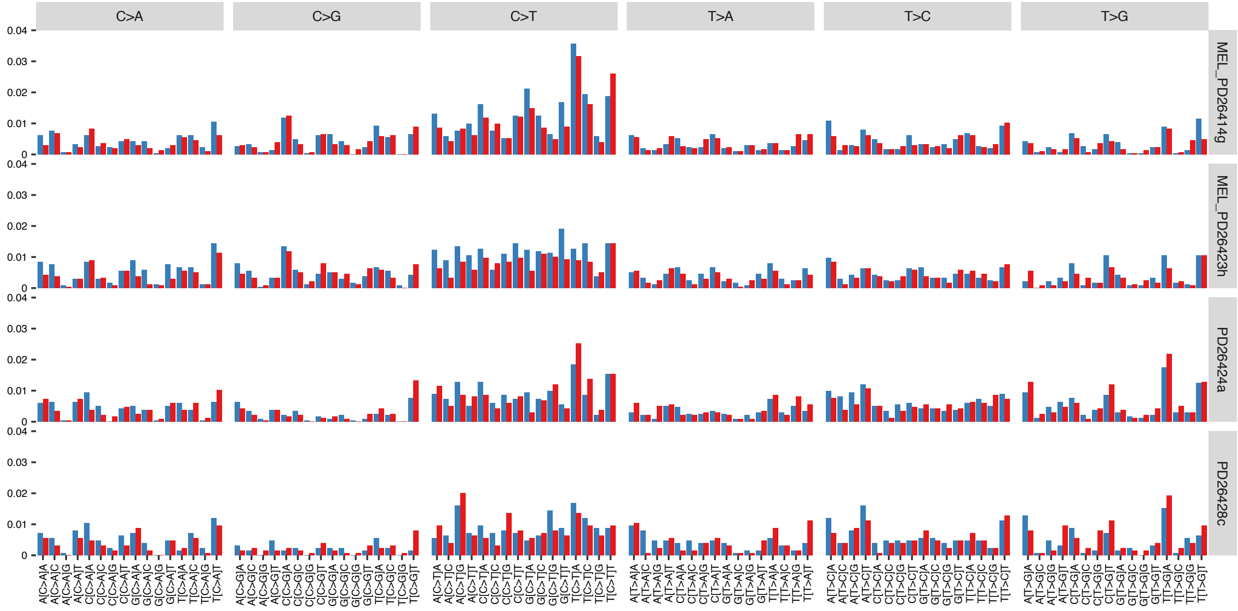

Figure S5: Localized hypermutation of the *IGH* locus of CLL patients with classical mutated and unmutated *IGHV*. The intermutational distance (y-axis) of mutations in the *IGH* locus (x-axis) are shown for illustrative patients. From top to bottom: **CLL56**, unmutated CLL with no evidence of AID activity; **CLL30**, unmutated CLL with localized hypermutation in the class-switch recombination (CSR) region only, sparing the coding V(D)J genes; **CLL1078**, unmutated CLL with evidence of extensive genome-wide AID activity, showing localized hypermutation in both the CSR and V(D)J region, though below the threshold for defining mutated CLL (i.e. >98 % conserved *IGHV*); **CLL373**, mutated CLL with evidence of extensive AID activity genome-wide as well as in the CSR and V(D)J regions, resulting in 87.8 % *IGHV* identity.

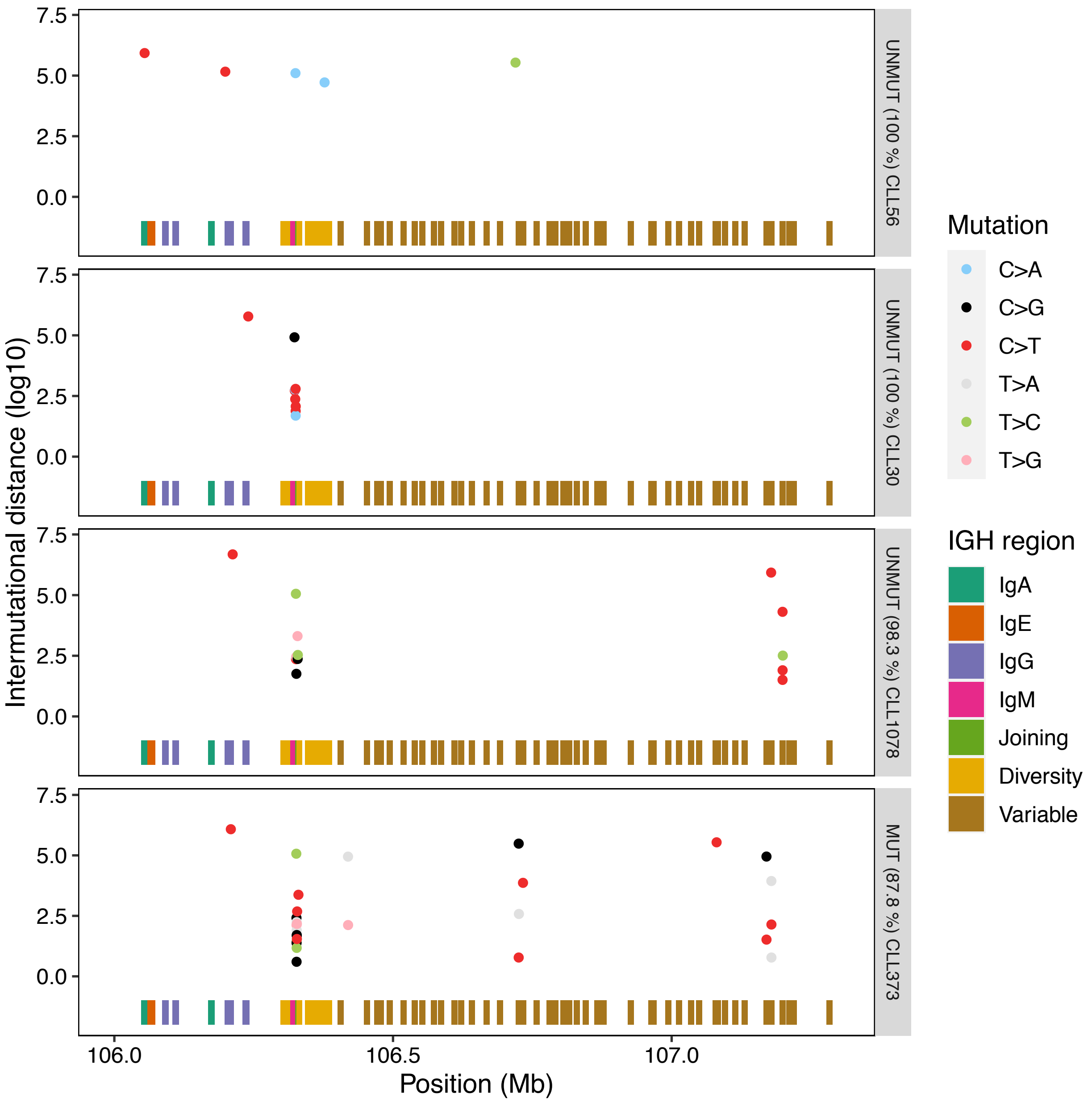

Supplement: Supplementary file 2 — Supplementary Information [file 42003_2021_1938_MOESM2_ESM.pdf]
